# Mouse Pancreas Tissue Slice Culture Facilitates Long-Term Studies of Exocrine and Endocrine Cell Physiology *in situ*

**DOI:** 10.1371/journal.pone.0078706

**Published:** 2013-11-04

**Authors:** Anja Marciniak, Claudia Selck, Betty Friedrich, Stephan Speier

**Affiliations:** 1 CRTD - DFG Research Center for Regenerative Therapies Dresden, Technische Universität Dresden, Dresden, Germany; 2 Paul Langerhans Institute Dresden, German Center for Diabetes Research (DZD), Dresden, Germany

## Abstract

Studies on pancreatic cell physiology rely on the investigation of exocrine and endocrine cells *in vitro*. Particularly, in the case of the exocrine tissue these studies have suffered from a reduced functional viability of acinar cells in culture. As a result not only investigations on dispersed acinar cells and isolated acini were limited in their potential, but also prolonged studies on pancreatic exocrine and endocrine cells in an intact pancreatic tissue environment were unfeasible. To overcome these limitations, we aimed to establish a pancreas tissue slice culture platform to allow long-term studies on exocrine and endocrine cells in the intact pancreatic environment. Mouse pancreas tissue slice morphology was assessed to determine optimal long-term culture settings for intact pancreatic tissue. Utilizing optimized culture conditions, cell specificity and function of exocrine acinar cells and endocrine beta cells were characterized over a culture period of 7 days. We found pancreas tissue slices cultured under optimized conditions to have intact tissue specific morphology for the entire culture period. Amylase positive intact acini were present at all time points of culture and acinar cells displayed a typical strong cell polarity. Amylase release from pancreas tissue slices decreased during culture, but maintained the characteristic bell-shaped dose-response curve to increasing caerulein concentrations and a ca. 4-fold maximal over basal release. Additionally, endocrine beta cell viability and function was well preserved until the end of the observation period. Our results show that the tissue slice culture platform provides unprecedented maintenance of pancreatic tissue specific morphology and function over a culture period for at least 4 days and in part even up to 1 week. This analytical advancement now allows mid -to long-term studies on the cell biology of pancreatic disorder pathogenesis and therapy in an intact surrounding *in situ*.

## Introduction

Malfunction of the exocrine or endocrine pancreas can lead to a number of devastating and life threatening diseases like pancreatitis, pancreatic cancer or diabetes mellitus. For a better understanding of the pathogenesis of these disorders detailed studies of pancreas function at a cellular level are indispensable and constitute a crucial step for the discovery of new treatment approaches. Type and characteristics of the cell preparations used in these studies determine which physiological and pathophysiological states can be simulated and which conclusions can be made for *in vivo* organ function. Most commonly primary exocrine acinar cells and endocrine beta cells are obtained as dispersed cells by enzymatic digestion of pancreatic tissue followed by exposure to chelating agents [1]. However, in the pancreas acinar cells and beta cells are organized in tissue specific structures, the acinus and the islet of Langerhans, respectively. Correct performance of both cell types has been shown to depend on cell to cell contacts established in these functional units [2], [3]. Thus, isolated acini and islets of Langerhans have been the preparation of choice when aiming to study pancreatic exocrine and endocrine cell biology in a close to physiological setting *in vitro*.

Islets of Langerhans can be easily isolated by subjecting the pancreas to collagenase digestion and mechanical shearing. Subsequently, islets can be maintained in culture for more than a week. However, it has been shown that the enzymatic isolation procedure causes fundamental structural changes and induces up-regulation of stress genes, a strong inflammatory response as well as increased production of reactive oxygen species and apoptosis [4], [5], [6], [7], [8]. Primary acini can be similarly isolated and examined *in vitro*. But also acini are sensitive to the enzymatic isolation procedure and have been reported to respond with stress-activated protein kinase activation, upregulation of cytokines and increased heat shock protein expression, resulting in functional changes [9], [10]. Additionally, freshly prepared acini degenerate and lose their native secretion characteristics within hours in culture [11]. Various attempts have aimed to prolong the experimental time period to study primary acinar cells *in vitro.* These approaches were able to prolong survival time of dispersed acinar cells in culture to several days, but could not maintain acini structure and function for more than 24 hours [11]. This lack of an acini preparation that is both responsive to a secretagogue and viable in long-term culture has been a major limitation for the study of disorders related to the exocrine pancreas [12], [13].

Acutely prepared mouse and rat pancreas tissue slices have been proven to be a valuable platform to study islet cell function *in situ* [14], [15], [16], [17]. In comparison to isolated acini and islets tissue slices feature several advantages including: 1) a short preparation time, 2) the lack of any added enzymatic disturbances, and 3) the preserved morphology of the pancreas. These characteristics of tissue slices enabled studying pancreatic islet cell biology under close to physiological conditions in a conserved environment and facilitated novel studies on mechanisms in health and disease [14], [15], [18], [19], [20]. In addition, pancreas tissue slices are most suitable for the investigation of acinar cell function, not only in intact individual acini, but in acini situated within the maintained lobular tissue structure of the pancreas. However, adult pancreas tissue slices experience loss of cell function and degeneration of cells within short time. We here developed a pancreas tissue slice culture platform which facilitates prolonged survival and function of the exocrine tissue while preserving the function of the endocrine component. We found that in optimized organotypic culture conditions pancreas tissue slices preserved tissue specific exocrine and endocrine morphology and function for 4 days and to a slightly lesser degree even up to 7 days. Therefore, this platform for the first time allows detailed long-term studies of cell physiology of pancreatic acini and islets within the intact pancreatic tissue *in situ*.

## Materials and Methods

### Preparation and organotypic culture of pancreas tissue slices

All animal experiments were conducted in a licensed animal facility in accordance with the German Animal Welfare Act, following the guidelines of the European Convention for the Protection of Vertebrate Animals Used for Experimental and Other Scientific Purposes and approved by the Committee on the Ethics of Animal Experiments of the State Directory of Saxony (24-916 8.21-1/2 009- 20 and 24-9168.24-1/2012-21).

Pancreas tissue slices from 8 – 16 weeks old C57Bl/6J and mouse insulin I promoter - green fluorescent protein (MIP-GFP) mice [21] of either sex were prepared as described previously [16]. Briefly, 1.25% low-melting point agarose in extracellular solution (ECS) consisting of (mmol/L): 140 NaCl, 5 KCl, 2 NaHCO_3_, 1 NaH_2_PO_4_, 1.2 MgCl_2_, 1.5 CaCl_2_, 3 glucose and 10 HEPES (pH 7.4 with NaOH) at 37°C was injected into the distally clamped bile duct. The injected pancreas tissue was cooled with ice-cold ECS and extracted. Small blocks of tissue (0.5 – 1.0 cm^3^ in size) were excised and embedded in agarose. Individual cubes of agarose containing the injected pancreas tissue were cut at 0.10 mm sec^−1^ and 70 Hz into 150 µm-thick slices using a vibratome (VT 1200, Leica, Germany). Preferentially tissue slices containing uncut intact islets were used for experiments. During and after slicing the tissue was kept in ice-cold ECS and used on the same day or placed on cell culture inserts (semipermeable membranes, pore size 0.4 µm, diameter 30 mm, Millipore, Ireland) for organotypic culture. In optimized conditions culture insert membranes were coated with a collagen gel mixture consisting of 3 mg/ml rat tail collagen type I, 1x phosphate buffered saline and 0.025 N NaOH. The standard medium consisted of RPMI-1640 with 5.5 mmol/L glucose, 10% fetal bovine serum, 20 mmol/L HEPES, and penicillin (100 U/ml)/streptavidin (100 µg/ml). The optimized medium was composed of Waymouth‘s MB 752/1 medium with 11 mmol/L glucose, 1% FBS, 0.1 mg/ml soybean trypsin inhibitor, 1 µg/ml dexamethasone, and penicillin (100 U/ml)/streptavidin (100 µg/ml). Cell culture inserts with slices were kept in 6 well plates filled with 1.1 ml of medium under a humidified atmosphere consisting of 95% air and 5% CO_2_ at 37°C. Culture at the air-medium interface was performed for at least 7 days and medium changed every 2 days. Cell viability was assessed using the dead cell marker Draq7 (Biostatus, UK) feasible for long-term culture. The dye was newly applied with every change of medium in a concentration of 3 µM.

### Stereomicroscopy and area measure

The morphology and surface area of slices in culture were monitored longitudinally using stereomicrography (SteREO Discovery.V12, Zeiss, Germany). Slice area was quantified manually in ImageJ by encircling the tissue borders.

### Immunohistochemistry

Immunohistochemistry was performed on freshly prepared slices after preparation and on cultured slices which had been detached from the culture insert membranes by mild shaking in ECS. Slices were fixed for 2 hours with 4% paraformaldehyde in PBS (pH 7.4) at 4°C C to stain against alpha-amylase or with a 7:3 methanol-acetone mixture at −20°C for anti-cytokeratin 19 (CK19) staining, and washed with 0.3% Triton-X100 in PBS at room temperature. Unspecific binding was blocked using 10% goat serum and 0.3% Triton-X100 in PBS for 1 hour at room temperature. Subsequently, slices were incubated with the primary antibody (50 µg/ml rabbit anti-alpha-amylase, Sigma, USA or 6 µg/ml rat anti-CK19, DSHB, USA) diluted with 1% goat serum and Triton-X100 in PBS for 16 hours at 4°C. After washing, incubation with the secondary antibody (20 µg/ml Alexa Fluor® 633 goat anti-rabbit IgG or 20 µg/ml Alexa Fluor® 633 goat anti-rat IgG, Molecular Probes, USA) followed for 2 hours at room temperature. DAPI (2.5 µg/ml, Sigma, USA) was applied with the secondary antibody to label nuclei. All incubation and washing steps were performed while shaking. Finally, slices were mounted using a Mowiol-based medium and examined by laser scanning confocal microscopy.

### Assessment of amylase and insulin secretion of pancreas tissue slices

Freshly prepared slices and cultured slices, which had been detached from the culture insert membranes by mild shaking in ECS, were rested for at least 2 hours in an incubation buffer consisting of ECS supplemented with 1 mg/ml bovine serum albumin, 0.1 mg/ml soybean trypsin inhibitor and 2 mmol/L L-glutamine. Basal and caerulein-induced amylase release of acute and cultured slices was determined by incubation in 500 µl incubation buffer without (basal secretion) or with the indicated concentrations of caerulein for 30 min at 37°C. Afterwards the supernatant was removed and slices were exposed to 500 µl of incubation buffer containing 3% Triton-X-100 for the assessment of slice amylase content. Supernatant and slice amylase content was measured with Liquid Amylase (CNPG3) Reagent Set (Pointe Scientific, USA). Basal and stimulated amylase secretion was expressed as percentage of total amylase content.

For measurement of glucose-stimulated insulin secretion acute and cultured slices were subsequently exposed to 500 µl incubation buffer containing low glucose (3 mmol/L), and high glucose (16.7 mmol/L) for 30 min at 37°C. Slice insulin content was extracted using acid ethanol (1.5% HCl, 70% ethanol) for 16 hours at −20°C. Insulin secreted in the supernatant and total insulin was determined by ultrasensitive mouse insulin enzyme-linked immunosorbent (ELISA) assay (Crystal Chem Inc, USA). Insulin release was expressed as percentage of total insulin content and the stimulation index calculated by dividing the insulin secreted upon stimulation by the insulin secreted in resting conditions.

### Confocal microscopy

Imaging was performed on a LSM 780 NLO (Zeiss, Germany) upright confocal microscope using a Plan-Apochromat 20x/1.0 water immersion objective. Fluorescence of MIP-GFP was excited at a wavelength of 488 nm and emission detected in the range of 500 − 600 nm. Alexa Fluor® 633 and Draq7 were excited at 633 nm and emission detected in the range of 650 − 750 nm. Backscattered laser light of 405 nm for morphological assessment was detected between 400 and 410 nm. The pinhole was adjusted to match the size of one airy unit. To obtain three-dimensional data image stacks with 2 µm step size were acquired. Islets of Langerhans and areas of exocrine tissue in pancreas slices were imaged longitudinally while kept on culture insert membrane and covered with PBS to allow immersion of the objective. Volume and cell number analysis was performed using Imaris v7.4.1 (Bitplane, Switzerland).

Changes in free intracellular calcium (Ca2+i) in acinar cells was monitored using the calcium indicator dye Oregon Green 488 BAPTA-1 AM calcium (OGB-1, Invitrogen, USA). Dye loading was performed as previously described [20]. In brief, slices were loaded in incubation buffer containing 6 µM OGB-1, 0.03% Pluronic F-127 (w/v), and 0.12% dimethylsulphoxide (v/v) for 1 hour at room temperature while shaking. After loading and before stimulation, the slices were stored in the incubation buffer for up to 6 hours at room temperature. OGB-1 was excited at 488 nm and emission detected in the range of 500 − 600 nm. The pinhole was adjusted to an optical section thickness of about 16 µm and images acquired every 2 s. Individual slices were stimulated using a temperature-controlled bath chamber (37°C, Warner Instruments, USA) and continuously perfused with 1.5 ml min^−1^ ECS containing indicated caerulein concentrations. Slices were held in place by slice anchors (Warner Instruments, USA).

### Statistical analysis

Data are presented as means ± SD. Slices used for analysis (n-number) were prepared from at least 3 different mice per experimental group. Statistical analysis of the data was performed using SPSS v21 (IBM, USA). Experimental groups were compared using a repeated or mixed measures ANOVA with p<0.05 considered as statistically significant (p<0.05 = *; p<0.01 = ** and p<0.005 = ***).

## Results

### Long-term preservation of slice morphology and surface area

**Figure 1:**
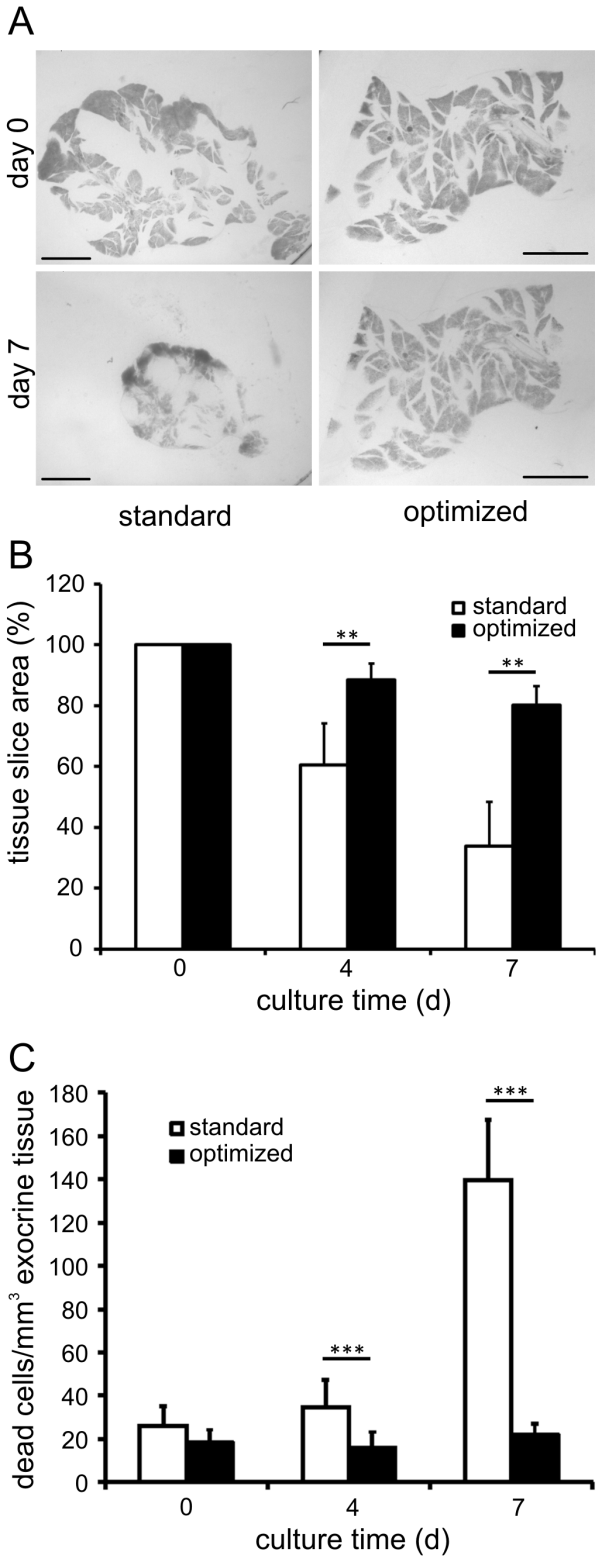
Preservation of pancreas tissue slice morphology under optimized organotypic culture conditions. *(A)* Panels show representative images of pancreas slices immediately after preparation and cultured for 7 days under standard and optimized conditions. Slices cultured in standard conditions exhibit dramatic changes of pancreas morphology whereas slices cultured in optimized conditions preserve the typical lobular structure of dense pancreatic tissue. Scale bars  =  2 mm. *(B)* Pancreas tissue slice area was maintained significantly better after 4 (88.5±5.3% vs. 59.6±19.2%) and 7 (80.2±6.2% vs. 33.1±15.7%) days when cultured under optimized conditions in comparison to standard conditions. Slice area values are expressed as percent of the area on the day of preparation and represent mean ± SD of n = 12 slices for optimized and n = 64 for standard conditions. (C) Quantification of Draq7 nuclei (dead cells) within exocrine tissue in freshly prepared pancreas tissue slices and during culture in standard and optimized conditions. Exocrine tissue volume was determined by backscatter LSM. Values are expressed as mean ± SD Draq7 labeled nuclei per mm^3^ exocrine tissue volume (n = 24 for optimized and n = 9 for standard conditions).

Tissue slices from adult mouse pancreas were cultured using an interface-membrane technique. Under conditions referred to as standard, freshly prepared pancreas slices of 150 μm thickness were placed on uncoated semipermeable membranes and cultured at the air-liquid interface employing a medium commonly used for the culture of isolated islets of Langerhans. Gross slice morphology and surface area were monitored using stereomicrography (Fig. 1A and B). On the day of preparation tissue slices displayed well-preserved pancreas morphology with lobular structures of exocrine cells containing pancreatic ducts and scattered islets. However, over a culture period of 7 days tissue slice area significantly decreased to 60.5±13.6% on day 4 and 33.8±14.6% on day 7, when compared to the initial surface area measured on day 0 (Fig. 1A). Morphology changed dramatically during this time period displaying loss and shrinkage of tissue, and disappearance of the typical pancreatic lobular structure (Fig. 1B). With the aim to prolong the culture period for intact pancreatic tissue, preservation of slice area and morphology were used to evaluate more than 50 culture conditions differing in basic culture medium, medium supplementation, serum concentration and substrate coating of the membrane (see Table S1 in the supporting information). However, the majority of conditions applied did not result in any obvious enhancement of slice area or morphology during long-term culture (data not shown).

Significant improvement of tissue preservation was achieved utilizing culture conditions based on an experimental setup employed for pancreas explant culture by Esni et al [22]. Therefore, semipermeable membranes were coated with rat tail collagen type I and a culture medium composed of Waymouth‘s MB 752/1 medium, soybean trypsin inhibitor, dexamethasone and penicillin/streptavidin was applied. In comparison to the previously reported media composition the formulation was adjusted to also allow maintenance of morphology and function of the endocrine component. The glucose concentration was lowered to 11 mmol/L to avoid effects of glucotoxicity and serum content was reduced to 1%, thereby prolonging islet cell viability (data no shown). These optimized culture conditions maintained slice morphology as well as surface area significantly better than standard conditions. Gross morphology of the tissue was well preserved even after 7 days of culture, displaying a conserved pancreatic lobular structure (Fig. 1B). Furthermore, residual tissue slice area on culture day 4 and 7 was significantly larger compared to standard conditions with 88.5±5.3% and 80.2±6.2% of initial slice area, respectively (Fig. 1A and B). This difference in conserved tissue slice area was due to an improved cell survival under optimized culture conditions. In freshly prepared tissue slices the number of dead cells per mm^3^ exocrine tissue was low with no significant difference between standard or optimized culture conditions (26.3±9.0 and 18.2±6.0 cells/mm^3^; Fig 1C). Whereas under optimized conditions the number of dead cells remained stable during culture (16.0±7.3 and 21.8±5.1 cells/mm^3^ on culture day 4 and day 7, respectively), the number of dead cells under standard conditions was significantly increased already at day 4 and further increased on day 7 of culture (34.7.0±12.6 and 139.7±27.8 cells/mm^3^ on culture day 4 and day 7, respectively, Fig. 1C).

### Amylase expression in pancreas exocrine tissue cells during long-term culture

**Figure 2:**
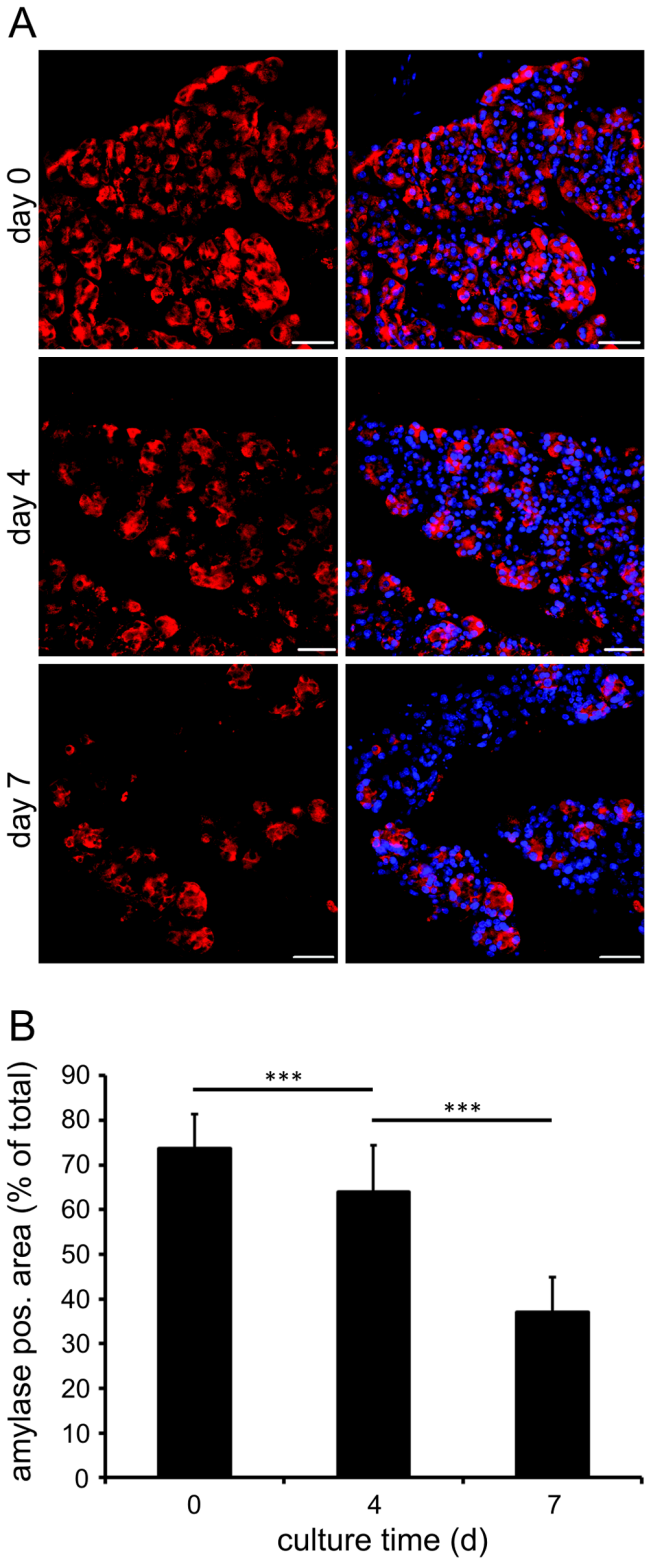
Effect of long-term pancreas tissue slice culture under optimized conditions on exocrine tissue morphology and amylase expression. *(A)* Immunohistochemistry for amylase (red) and DAPI (blue) in lobules of freshly prepared and under optimized conditions cultured pancreas tissue slices. Labeling revealed dense localization of amylase positive acini at day 0, occupying most of the pancreas lobule area (73.7±7.6%). Amylase labeled acini density and amylase positive area of lobules decreases, however, amylase positive acini are still present at day 4 and 7 of tissue slice culture, (63.9±10.5% and 37.1±7.9%; at day 4 and 7, respectively). Scale bars  = 50 µm. *(B)* Amylase positive lobule area in fresh and under optimized conditions cultured pancreas tissue slices at indicated time points. Values represent mean ± SD of 16 lobules in 8 slices per time point (n = 16).

For a more detailed assessment of exocrine tissue preservation acute slices and tissue slices cultured in optimized conditions were investigated by immunohistochemical staining for alpha-amylase expression. In freshly prepared pancreas slices lobules displayed a compact arrangement of amylase positive acini (Fig. 2A). Quantification revealed 73.7±7.6% of the lobule area positive for alpha-amylase staining at day 0 (Fig. 2B). During prolonged culture acini density decreased and lobules showed increasing amounts of amylase negative tissue (Fig. 2A). This was reflected by a gradual loss of amylase positive slice area until day 4 (63.9±10.5%) followed by a more prominent decrease until day 7 (37.1±7.9%). Notably, even on culture day 7 many amylase positive acini were detected within pancreas lobules (Fig. 2B).

### Long-term preservation of exocrine cell morphology and specificity

**Figure 3:**
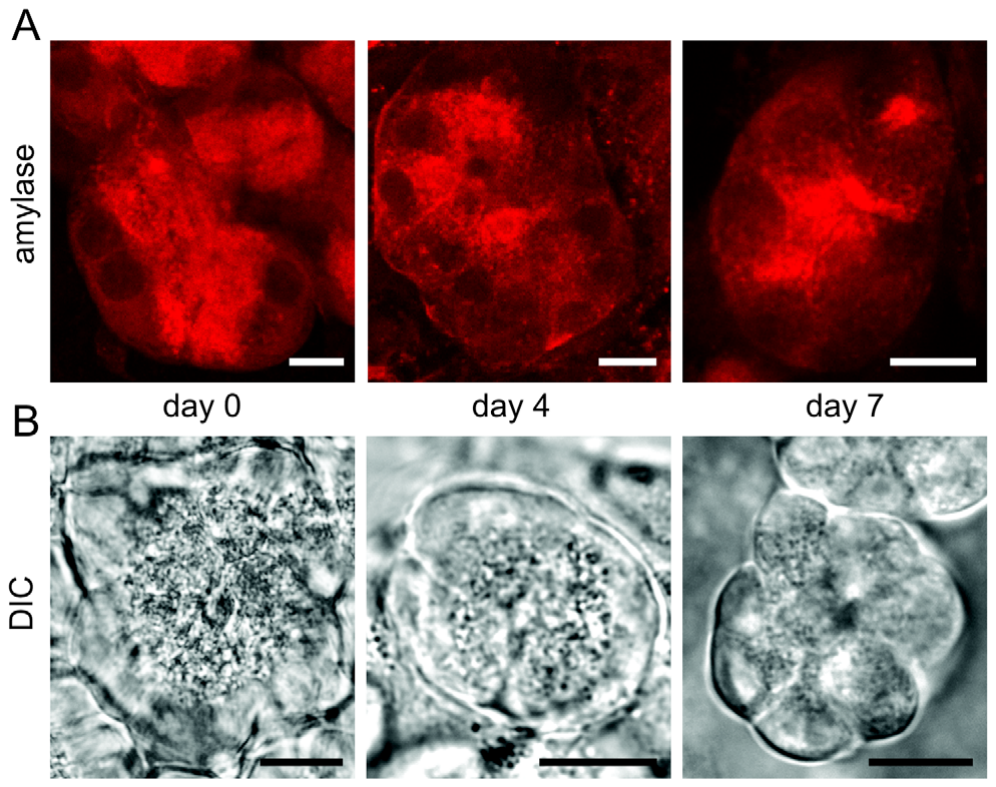
Cellular specificity and morphology of exocrine cells in long-term culture of pancreas tissue slices. *(A)* Cellular localization of amylase labeling in acini of tissue slices after preparation and on day 4 and 7 of optimized long-term culture. Immunohistochemistry showed polarized localization of amylase labeling at the apical acinar cell pole at all time points. *(B)* Differential interference contrast (DIC) microscopy of acini in tissue slices at identical time points as in (A). DIC microscopy revealed the presence of zymogen granules at the apical pole and a transparent basal pole of acinar cells in acini of pancreas slices right after preparation and at day 4 and 7 of tissue slice culture. Scale bars  = 10 µm.

Exocrine cell morphology and specificity in cultured slices was further evaluated by assessing preservation of the typical highly polarized acinar cell phenotype. Therefore, the distribution of alpha-amylase in individual acini was investigated using high resolution laser scanning microscopy of amylase stained acini within slices (Fig. 3A). On the day of preparation acinar cells demonstrated a clearly divided staining pattern with amylase labeling being more intense at the apical plasma membrane and less prominent at the basolateral cell pole. This distinctive pattern of alpha-amylase expression was observed in almost all amylase positive acini throughout the entire culture period.

Additionally, acinar cell specificity was verified at high resolution with differential interference contrast (DIC) microscopy (Fig. 3B). Like amylase staining, DIC microscopy of acini in tissue slices revealed cell polarity with granules located at the apical pole and a more transparent basal pole of the cells. This polarity could be observed in acini of acute tissue slices and in slices cultured for 4 and 7 days. Furthermore, slices were stained for the ductal cell marker CK19 to assess a possible transdifferentiation of acinar to ductal cells in culture. Surprisingly, a fine filamentous staining could be observed already in acinar cells of freshly prepared slices, which was not detectable in cryosections of pancreas. This filamentous staining was increased in intensity and density at culture day 4 and 7 throughout most acini of the tissue slices (data not shown).

### Long-term maintenance of exocrine secretory function

**Figure 4:**
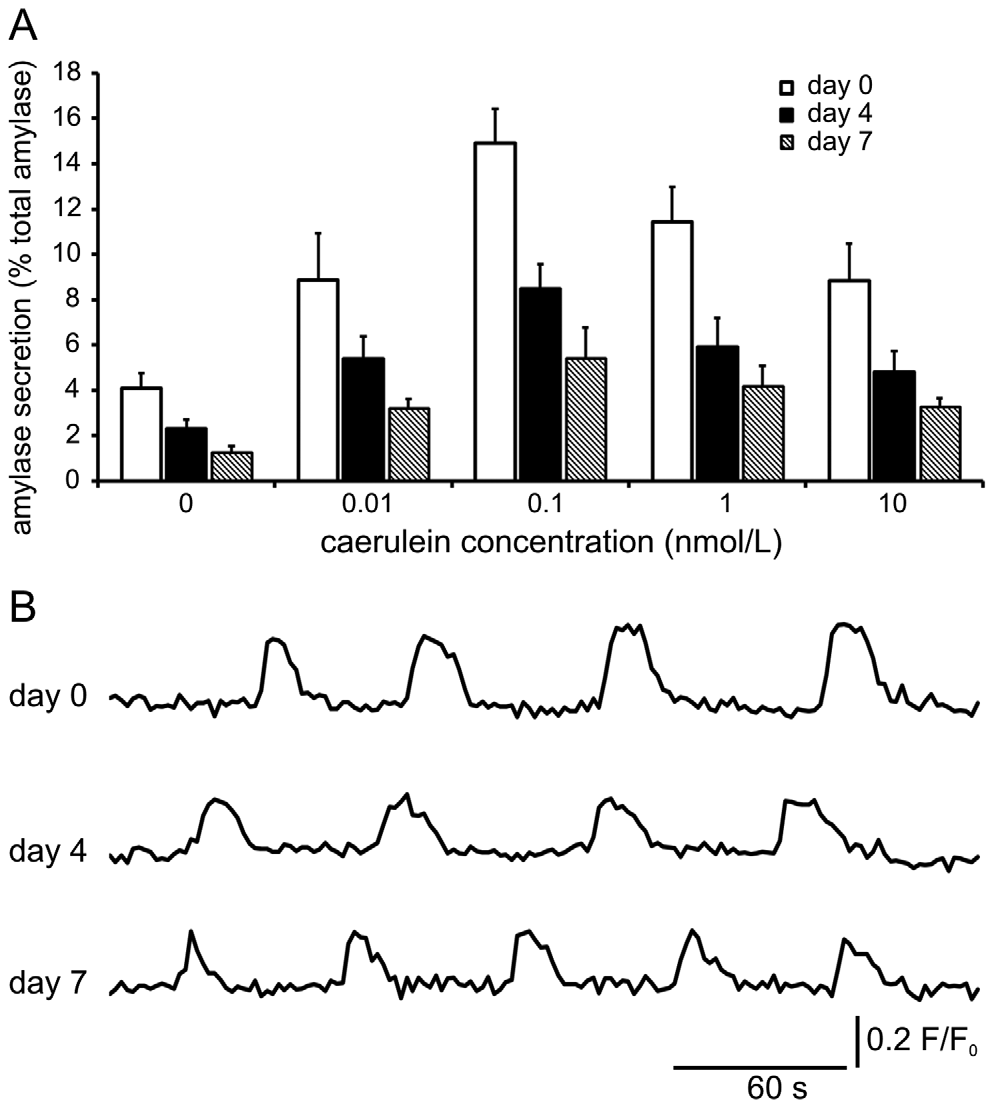
Acinar cell function during long-term culture of pancreas tissue slices. *(A)* Amylase release from freshly prepared and cultured pancreas tissue slices after 30 min stimulation with indicated caerulein concentrations. The relative amount of released amylase decreases with culture time. However, amylase release shows a typical bell-shaped curve response to increasing caerulein concentrations at all time points. Amylase release is expressed as percent of total amylase as mean ± SD of 16 slices per time point (n = 16). *(B)* Traces of Ca^2+^_i_/Oregon Green BAPTA-1 fluorescence in acinar cells of pancreas tissue slices at indicated time points after preparation. Stimulation with 10 pmol/L caerulein induced oscillations of Ca^2+^_i_/Oregon Green BAPTA-1 fluorescence in acinar cells of slices at all time points. Traces are shown as fluorescence ratio (F/F_0_), in which F is fluorescence at any given time and F_0_ is pre-stimulatory fluorescence.

Cell function of acinar cells in pancreas tissue slices was evaluated on the level of secretagogue-induced amylase release and changes in Ca^2+^_i_ (Fig. 4A and B). First, amylase secretion from pancreas tissue slices was measured at basal conditions without stimulation and after incubation with caerulein, a known secretagogue of amylase release, in concentrations of 0.01, 0.1, 1 and 10 nmol/L. On the day of preparation, tissue slices demonstrated a basal amylase release of 4.1±0.7% of total amylase content. Stimulation with rising concentrations of caerulein resulted in a typical bell-shaped curve (Fig. 4A). Maximal amylase release of 14.9±1.5% (ca. 4-fold of basal secretion) was measured after challenge with 0.1 nmol/L caerulein. A further increase in the caerulein concentration to 10 nmol/L resulted in supramaximal inhibition of amylase release to 8.8±1.6%. No increase in basal amylase secretion indicating acinar cell leakage was observed from pancreas slices cultured for 4 or 7 days. In contrast, the amount of amylase secreted under basal conditions decreased to 2.3±0.4% of total amylase content at day 4 and 1.2±0.3% at day 7. Accordingly, also the percentage of stimulated amylase release decreased in cultured slices. Maximal amylase release from tissue slices on day 4 and 7 was 8.5±1.1% and 5.4±1.4%, respectively, when stimulated with 0.1 nmol/L caerulein. Importantly, although the fraction of total amylase content secreted under basal and stimulatory conditions after four and seven days of culture decreased, amylase release sustained a bell-shaped curve response to increasing concentrations of caerulein and stimulated maximal release was ca. 4-fold basal secretion at all time points.

To address exocrine function at a cellular level changes in Ca^2+^_i_ after stimulation were investigated. Acute and cultured pancreas slices were loaded with a calcium sensitive indicator dye and changes in fluorescence intensity after stimulation determined by laser scanning microscopy. After application of 10 pmol/L caerulein similar oscillatory changes of Ca^2+^_i_ could be observed in acinar cells in acute slices and at day 4 and 7 after preparation in cultured slices (Fig. 4B and Video S1-S3 in the supporting information). Though, especially by day 7 the number of labeled but unresponsive acini had increased. Imaging at lower resolution enabled the simultaneous observation of larger areas within pancreatic lobules showing widespread activity of acini after stimulation (Video S1-S3 in the supporting information). In contrast, supramaximal inhibitory concentrations (10 nmol/L) of caerulein caused a global and prolonged Ca^2+^_i_ increase at all time points (Data not shown). The preservation of these characteristic calcium responses of acinar cells to maximal and supramaximal stimulation with caerulein, illustrates a conserved acinar cell function in acute and cultured tissue slices.

### Long-term preservation of islet cell viability and function

**Figure 5:**
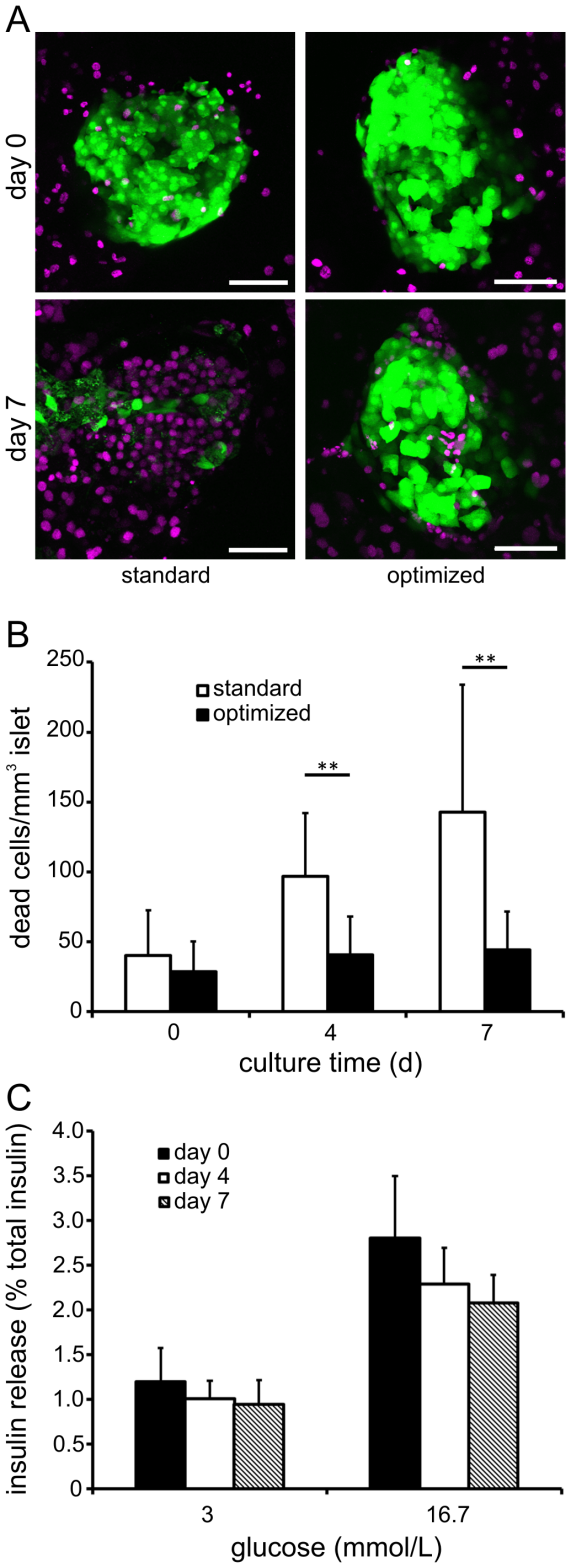
Effect of long-term pancreas tissue slice culture on endocrine beta cell viability and function. (*A*) Longitudinal *in situ* imaging of beta cell viability and specificity in pancreas tissue slices before and after 7 days culture in standard and optimized conditions. Under standard culture conditions MIP-GFP fluorescence (green) of beta cells was lost after 7 days and the numbers of dead nuclei (magenta) dramatically increased, whereas in optimized conditions beta cells were still detectable by GFP fluorescence after 7 days and the number of dead cells inside the islet remained low. *(B)* Quantification of Draq7 nuclei (dead cells) within islets in freshly prepared pancreas tissue slices and during culture in standard and optimized conditions. Islet volume in tissue slices was determined by backscatter LSM. Values are expressed as mean ± SD Draq7 labeled nuclei per mm^3^ islet volume (n = 11 for optimized and n = 12 for standard conditions). *(C)* Basal (3 mmol/L glucose) and stimulated (16.7 mmol/L glucose) insulin release from pancreas tissue slices at the day of preparation and after 4 and 7 days of culture in optimized conditions. Insulin release is expressed as percent of total insulin as mean ± SD from 8 slices per time point (n = 8).

To assess if our culture conditions would allow simultaneous investigations of the exocrine and endocrine pancreas we evaluated viability and function of the islets of Langerhans during the culture period of 7 days. Beta-cell specificity and islet morphology in culture were investigated using pancreas slices from MIP-GFP mice. Islet cell death was quantified by determining dead cells within the islet volume in 3D. In freshly prepared slices beta-cells demonstrated an intensive GFP fluorescence indicating existing activity of the mouse insulin reporter (Fig. 5A). Furthermore, only few dead islet cells were observed on the day of preparation with no significant difference between standard or optimized culture conditions (40.3±32.4 and 28.5±21.7 cells/mm^3^ islet; Fig 5A and B). However, during prolonged culture using standard conditions an increasing number of dead nuclei were detected within the islet (96.8±45.5 and 142.8±91.0 cells/mm^3^ islet on culture day 4 and day 7, respectively) (Fig. 5A and B). Additionally, disappearance of GFP fluorescence within the islets during culture indicated beta cell death and loss of insulin promoter activity. In contrast, in slices cultured in optimized conditions long-standing GFP fluorescence revealed preserved viability and specificity of beta cells for at least 7 days. Under these conditions islets displayed stable morphology with low numbers of dead islet cells (40.5±27.6 and 44.1±27.7 cells/mm^3^ islet on culture day 4 and day 7, respectively) (Fig. 5A and B).

Endocrine secretory function of beta cells in pancreas tissue slices was assessed by measurement of insulin release at basal (3 mmol/L) and stimulating (16.7 mmol/L) glucose concentrations. Freshly prepared pancreas slices demonstrated a basal release of 1.2±0.4% of total insulin which increased to 2.8±0.7% after stimulation with 16.7 mmol/L glucose, indicating that the intact islet capsule in slices did not interfere with glucose stimulation or insulin release. Similarly to amylase secretion, basal and stimulated insulin release from beta cells was slightly decreased after 4 (1.0±0.2% vs. 2.3±0.4%) and 7 days (0.9±0.3% vs. 2.1±0.3%) in culture, but the stimulation index, as an indicator of beta cell function, was similar at all time points with 2.4±0.3, 2.3±0.3 and 2.3±0.4, respectively. Thus, regulated insulin release was maintained during long-term tissue slice culture.

## Discussion

We here have successfully established and characterized an organotypic culture system for adult mouse pancreas tissue slices, enabling long-term studies on exocrine and endocrine cell physiology *in situ*. Using optimized culture conditions endocrine beta cells and exocrine acinar cells remained morphologically intact and exhibited their characteristic secretory function for a prolonged time period of 4 days. Even though to a lesser extent, morphologically and functionally intact acini could still be found in cultured slices at day 7 after preparation. This was achieved by employing an interface-membrane technique with pancreas slices placed on culture insert membranes providing a stable substrate essential for preservation of slice morphology. Furthermore, coating with a collagen mixture allowed long-term preservation of the typical pancreas morphology which could not be achieved with other tested coatings (gelatin or Matrigel™, see Table S1 in the supporting information). Additionally, we evaluated several medium environments differing in basic culture medium and medium supplementation (see Table S1 in the supporting information). Best results for tissue preservation were achieved when using a medium composition based on conditions previously employed for pancreas explant cultures [22], but modified to meet the specific requirements of the endocrine component. We used custom made, glucose free Waymouth’s MB 752/1 medium to allow reduction of glucose content from 28 mmol/L to more physiological concentrations to prevent beta-cell glucotoxicity [23], [24]. Detailed characterization of exocrine and endocrine cell viability as well as secretory function demonstrated that glucose levels as high as 28 mmol/L were not essential for long-term culture of pancreatic tissue slices and revealed 11 mmol/L glucose as the most suitable concentration in respect to beta cell survival and glucose induced insulin release (data not shown). Furthermore, we lowered FBS content from 10 to 1% which prolonged islet cell viability (data not shown) and minimized the ambiguous influence of FBS on acinar and beta cell physiology. Thus, we present an optimized culture medium avoiding high serum and glucose concentrations, and omitting addition of growth factors or secretagogues, which enables combined studies on exocrine and endocrine cell physiology in a well-defined and physiological setting *in situ*.

Utilizing the established optimized culture conditions we characterized the preservation of specificity and function of acinar and beta cells in long-term culture of pancreas tissue slices. Detailed analysis of exocrine tissue preservation showed only a slight reduction of amylase-positive area during the first 4 days of culture to about 90% of the amylase positive area observed at day 0. This was followed by a more prominent decrease to about 50% of original amylase positive area at culture day 7, indicating loss of amylase expression in parts of the exocrine tissue during prolonged culture periods. Nevertheless, numerous intact amylase positive acini were detected in lobules of pancreas tissue slices even after 7 days of culture. Subsequently we investigated the specificity of acinar cells in cultured slices by assessing cell morphology and cellular distribution of amylase staining. A loss of the characteristic apical–basal polarity of acinar cells with amylase containing zymogen granules located at the apical cell pole is an early sign of cell degeneration and dedifferentiation and occurs after 24 hours culture of isolated acini [11]. However, in the current study we observed highly polarized acinar cells in pancreas tissue slices after 4 and 7 days of culture, with zymogen granules and amylase expression predominantly at the apical plasma membrane. Preservation of cellular specificity of acinar cells in pancreas tissue slices was supported by their sustained secretory function. Pancreatic acinar cells in freshly prepared and cultured tissue slices demonstrated a typical bell-shaped dose-response curve for amylase secretion when stimulated with increasing concentrations of caerulein. Although, absolute amounts of amylase release from slices decreased with culture time, the ratio of stimulated over basal release was comparable at all time points, demonstrating an intact regulated secretion. In addition, intact cell physiology for up to 7 days of culture was illustrated by the observation of typical oscillatory Ca^2+^_i_ changes in individual acini in response to physiological concentrations of caerulein. Thus, morphology, polarity and function indicate a sustained differentiated state of acinar cells during culture. However, the observed increase of CK19 staining in amylase positive and polarized acinar cells might point to a beginning acinar-to-ductal transdifferentiation, which has not led to any morphological or functional changes to that time point, but should be considered, especially in experiments exceeding the here characterized culture period.

Finally, well preserved islet of Langerhans/beta cell viability and function during the entire observation period supports the suitability of pancreas tissue slice culture as a model for long-term *in situ* studies on intact pancreatic tissue.

The beneficial preparation procedure and intact tissue specific morphology make pancreas tissue slices an excellent approach to study cell physiology of acinar and beta cells in an *in situ* environment. In addition, the here presented long-term organotypic culture system for pancreas tissue slices now provides a unique technique to use this approach for longitudinal studies on pancreatic exocrine and endocrine cells under near-physiological conditions. Therefore, this technical platform enables addressing various aspects of pancreas physiology. The prolonged experimental time for studying functional and viable acini in culture simplifies the use of tools for cell manipulation and extends the observation period after compound application. Importantly, pancreas tissue slice culture also accelerates detailed investigations of pancreatic disorders. In combination with the available mouse models for pancreatic cancer [25]_ENREF_30, acute and chronic pancreatitis [26], [27] as well as type 1 and type 2 diabetes [28] the extended time period benefits investigations of cellular mechanisms and intervention *in situ*. Future studies will show if pancreas tissue slice culture also opens up new possibilities for the establishment of appropriate *in situ* models of pancreatic diseases.

Finally, the presence of both, pancreatic exocrine and endocrine tissue in a close to physiological environment for long-term observations facilitates investigations of processes regarding cell interactions in health and disease [29], [30], and cell transdifferentiation during regeneration [31], [32], [33]. The possibility of simultaneous long-term studies will also allow addressing the effect of compounds on either tissue under identical conditions. Therefore, pancreas tissue slice culture might help to shed light on controversial discussions about possible negative side effects of anti-diabetic drugs on the exocrine pancreas, e.g. glucagon like peptide-1 analogues [34], [35], [36].

## Supporting Information

Table S1 The table lists the majority of culture conditions tested differing in basic culture medium, medium supplementation, serum concentration and substrate coating of the culture insert membrane, including the standard (#1) and optimized condition (#50).
(PDF)

Video S1 **Ca^2+^_i_/Oregon Green BAPTA-1 fluorescence in acinar cells of a pancreas tissue slice at the day of preparation before and after stimulation with 10 pM caerulein at 60 s.** Acini within tissue slices show widespread Ca^2+^_i_/Oregon Green BAPTA-1 fluorescence activity throughout the entire lobule. Fluorescence intensity is displayed as Rainbow RGB lookup table with low fluorescence intensities (low Ca^2+^_i_ in blue), over medium fluorescence intensities in green to high fluorescence intensities (high Ca^2+^_i_ in red). Sampling frequency  =  0.5 Hz. Scale bar  =  50 µm.
(MOV)

Video S2 **Ca^2+^_i_/Oregon Green BAPTA-1 fluorescence in acinar cells of a pancreas tissue slice after 4 days culture in optimized conditions and after stimulation with 10 pM caerulein.** As on day 0 acini within tissue slices show widespread Ca^2+^_i_/Oregon Green BAPTA-1 fluorescence activity throughout the entire lobule. Fluorescence intensity is displayed as Rainbow RGB lookup table with low fluorescence intensities (low Ca^2+^_i_ in blue), over medium fluorescence intensities in green to high fluorescence intensities (high Ca^2+^_i_ in red). Sampling frequency  =  0.5 Hz. Scale bar  =  50 µm.
(MOV)

Video S3 **Ca^2+^_i_/Oregon Green BAPTA-1 fluorescence in acinar cells of a pancreas tissue slice after 7 days culture in optimized conditions and after stimulation with 10 pM caerulein.** In comparison to day 4 less acini respond to stimulation by changes in fluorescence. However, still widespread Ca^2+^_i_/Oregon Green BAPTA-1 fluorescence activity of functional acini throughout the entire lobule can be detected. Fluorescence intensity is displayed as Rainbow RGB lookup table with low fluorescence intensities (low Ca^2+^_i_ in blue), over medium fluorescence intensities in green to high fluorescence intensities (high Ca^2+^_i_ in red). Sampling frequency  =  0.5 Hz. Scale bar  =  50 µm.
(MOV)
