# Supplementary material for: Mouse Pancreas Tissue Slice Culture Facilitates Long-Term Studies of Exocrine and Endocrine Cell Physiology in situ
Source: PLoS One. 2013 Nov 4;8(11):e78706. doi: 10.1371/journal.pone.0078706 (PMC3817072; doi:10.1371/journal.pone.0078706)
Supplement: Table S1 — The table lists the majority of culture conditions tested differing in basic culture medium, medium supplementation, serum concentration and substrate coating of the culture insert membrane, including the standard (#1) and optimized condition (#50). (PDF) [file pone.0078706.s001.pdf]

Table S1. Culture conditions tested for pancreas slice culture

Condition	Basic culture medium	Medium supplementation	Serum concentration	Glucose concentration	Substrate coating
**1 (Standard)**	RPMI-1640	/	10 %	5.5 mM	/
**2**	CMRL-1066 (- L-glut)	/	10 %	5.5 mM	/
**3**	CMRL-1066 (+ L-glut)	/	10 %	5.5 mM	/
**4**	Ham's F-12	/	10 %	5.5 mM	/
**5**	Waymouth's MB 752/1	/	10 %	5.5 mM	/
**6**	MEM	/	10 %	5.5 mM	/
**7**	DMEM	/	10 %	5.5 mM	/
**8**	RPMI-1640	/	10 %	3.0 mM	/
**9**	RPMI-1640	/	10 %	11.0 mM	/
**10**	RPMI-1640	0.1 mg / ml STI	10 %	5.5 mM	/
**11**	RPMI-1640	0.2 mg / ml STI	10 %	5.5 mM	/
**12**	RPMI-1640	0.4 mg / ml STI	10 %	5.5 mM	/
**13**	RPMI-1640	1x ITS-A	10 %	5.5 mM	/
**14**	RPMI-1640	2x ITS-A	10 %	5.5 mM	/
**15**	RPMI-1640	4x ITS-A	10 %	5.5 mM	/
**16**	RPMI-1640	100 µM Carbachol	10 %	5.5 mM	/
**17**	RPMI-1640	10 nM Caerulein	10 %	5.5 mM	/
**18**	RPMI-1640	/	/	5.5 mM	/
**19**	RPMI-1640	/	1 %	5.5 mM	/
**20**	RPMI-1640	/	5 %	5.5 mM	/
**21**	RPMI-1640	/	15 %	5.5 mM	/
**22**	RPMI-1640	/	20 %	5.5 mM	/
**23**	RPMI-1640	/	10 %	5.5 mM	Matrigel™
**24**	RPMI-1640	/	10 %	5.5 mM	3% gelatin
**25**	RPMI-1640	/	10 %	5.5 mM	3 mg / ml collagen
**26**	RPMI-1640	0.2 mg / ml STI	10 %	5.5 mM	3 mg / ml collagen
**27**	RPMI-1640	/	1 %	5.5 mM	3 mg / ml collagen
**28**	RPMI-1640	/	1 %	11.0 mM	3 mg / ml collagen
**29**	RPMI-1640	/	1 %	16.7 mM	3 mg / ml collagen
**30**	RPMI-1640	/	1 %	28.0 mM	3 mg / ml collagen
**31**	RPMI-1640	200 x Protease Inhibitor Cocktail	1 %	5.5 mM	3 mg / ml collagen
**32**	RPMI-1640	0.1 mg / ml STI	1 %	5.5 mM	3 mg / ml collagen
**33**	RPMI-1640	0.1 mg / ml STI<br>1 µg / ml dexamethasone	1 %	5.5 mM	3 mg / ml collagen

**34**	RPMI-1640	0.1 mg / ml STI<br>1 µg / ml dexamethasone	1 %	28.0 mM	3 mg / ml collagen
**35**	Waymouth's MB 752/1	0.1 mg / ml STI<br>1 µg / ml dexamethasone	1 %	28.0 mM	3 mg / ml collagen
**36**	Waymouth's MB 752/1	0.2 mg / ml STI<br>1 µg / ml dexamethasone	1 %	28.0 mM	3 mg / ml collagen
**37**	Waymouth's MB 752/1	0.1 mg / ml STI	1 %	28.0 mM	3 mg / ml collagen
**38**	Waymouth's MB 752/1	/	1 %	28.0 mM	3 mg / ml collagen
**39**	Waymouth's MB 752/1	0.1 mg / ml STI<br>1 µg / ml dexamethasone<br>200 x Protease Inhibitor Cocktail	1 %	28.0 mM	3 mg / ml collagen
**40**	Waymouth's MB 752/1	200 x Protease Inhibitor Cocktail	1 %	28.0 mM	3 mg / ml collagen
**41**	Waymouth's MB 752/1	0.1 mg / ml STI<br>1 µg / ml dexamethasone<br>50 µM β-Mercaptoethanol	1 %	28.0 mM	3 mg / ml collagen
**42**	Waymouth's MB 752/1	0.1 mg / ml STI<br>1 µg / ml dexamethasone	/	28.0 mM	3 mg / ml collagen
**42**	Waymouth's MB 752/1	0.1 mg / ml STI<br>1 µg / ml dexamethasone	5 %	28.0 mM	3 mg / ml collagen
**43**	Waymouth's MB 752/1	0.1 mg / ml STI<br>1 µg / ml dexamethasone	10 %	28.0 mM	3 mg / ml collagen
**44**	Waymouth's MB 752/1	0.1 mg / ml STI<br>1 µg / ml dexamethasone	10 %	28.0 mM	/
**45**	Waymouth's MB 752/1	0.1 mg / ml STI<br>1 µg / ml dexamethasone	1 %	28.0 mM	2 mg / ml collagen
**46**	Waymouth's MB 752/1	0.1 mg / ml STI<br>1 µg / ml dexamethasone	1 %	28.0 mM	4 mg / ml collagen
**47**	Waymouth's MB 752/1	0.1 mg / ml STI<br>1 µg / ml dexamethasone	1 %	28.0 mM	/
**48**	Waymouth's MB 752/1	0.1 mg / ml STI<br>1 µg / ml dexamethasone	1 %	28.0 mM	2 mg / ml fibrin
**49**	Waymouth's MB 752/1	0.1 mg / ml STI<br>1 µg / ml dexamethasone	1 %	5.5 mM	3 mg / ml collagen
**50 (Optimized)**	Waymouth's MB 752/1	0.1 mg / ml STI<br>1 µg / ml dexamethasone	1 %	11.0 mM	3 mg / ml collagen
**51**	Waymouth's MB 752/1	0.1 mg / ml STI<br>1 µg / ml dexamethasone	1 %	16.7 mM	3 mg / ml collagen
**52**	Waymouth's MB 752/1	0.1 mg / ml STI<br>1 µg / ml dexamethasone	1 %	11.0 mM	2 mg / ml fibrin
